# Clinician‐reported chorionicity and zygosity assignment using single‐nucleotide polymorphism‐based cell‐free DNA: Lessons learned from 55,344 twin pregnancies

**DOI:** 10.1002/pd.6218

**Published:** 2022-09-07

**Authors:** Anna Wojas, Kimberly A. Martin, Allyson Koyen Malashevich, Katelyn Hashimoto, Sheetal Parmar, Roseann White, Zachary Demko, Paul Billings, Russ Jelsema, Andrei Rebarber

**Affiliations:** ^1^ Department of Obstetrics, Gynecology, and Reproductive Science Mt. Sinai New York NY USA; ^2^ Natera Inc Austin Texas USA

## Abstract

**Objective:**

Prenatal chorionicity assessment relies on ultrasound, which can be confounded by many factors. Noninvasive assessment of zygosity is possible using single nucleotide polymorphism (SNP)‐based cell‐free DNA testing. Our objective was to determine the relationship between provider‐reported chorionicity and SNP‐cfDNA assignment of twin zygosity.

**Methods:**

All twin pregnancy blood samples received by a reference laboratory between September 27, 2017 and September 8, 2021 were included. Chorionicity assignment was requested on the requisition, recorded as; monochorionic (MC), dichorionic, or “don't know”. SNP‐cfDNA zygosity results, monozygotic (MZ) or dizygotic (DZ), were correlated with chorionicity assignment.

**Results:**

59,471 twin samples (median gestational age = 12.0 weeks at draw) were received and analyzed; 55,344 (93.1%) received zygosity assignment. SNP‐cfDNA reported 16,673 (30.1%) MZ and 38,671 (69.9%) as DZ. Provider‐reported chorionicity was compared to the zygosity assignment for each case. Of 6283 provider‐reported MC twins, 318 (5.1%) were reported as DZ using SNP‐cfDNA.

**Conclusion(s):**

One in 20 suspected MC twin pregnancies were reported as DZ using SNP‐cfDNA. Approximately 30% of 55,344 twin pregnancies were found to be MZ, including cases where chorionicity was unknown. SNP‐cfDNA zygosity assessment is a useful adjunct assessment for twin pregnancies, particularly those reported as MC or without determined chorionicity.

## Key points

What's already known about the topic?

- The assignment of chorionicity early in pregnancy can improve perinatal outcomes of twin pregnancies. Prenatal assessment of chorionicity relies on ultrasound (US), and the accuracy of US can be confounded by many factors, including gestational age (GA) and operator experience. Noninvasive assessment of zygosity is now possible using single nucleotide polymorphism (SNP)‐based cell‐free DNA (cfDNA).

What does this study add?

- This is the first population‐based study describing the correlation between cfDNA assessment of zygosity and chorionicity. In 55,344 twin pregnancies, approximately 30% were found to be monozygotic (MZ), including cases where chorionicity was unknown. We found that one in 20 suspected monochorionic (MC) twin pregnancies were reported as dizygotic (DZ) twins (DZ) by SNP‐cfDNA testing. SNP‐cfDNA zygosity assessment is a useful adjunct assessment for twin pregnancies, particularly those reported as MC or without determined chorionicity.

## 1 INTRODUCTION

Twin gestations account for approximately 1 in 32 live births in the United States.^1^ Compared to singletons, twins have higher rates of perinatal complications, which include miscarriage, birth defects, preterm birth, gestational hypertension, gestational diabetes, abnormalities in amniotic fluid volume, intrauterine fetal growth restriction, maternal anemia, postpartum hemorrhage, and cesarean section.^2^ Chorionicity, the distribution of the placental membranes, is a major factor impacting the prognosis for twin pregnancies. A 2013 study by D’Antonio et al.^3^ determined that MC twins have increased rates of early fetal loss (10x higher at GA less than 24 weeks) and perinatal mortality (2x higher), as compared to dichorionic (DC) twins. As indicated by Lopriore et al.,^4^ MC twins are at increased risk for twin‐twin transfusion syndrome (TTTS), twin anemia polycythemia sequence, and selective fetal growth restriction. The assignment of chorionicity early in pregnancy can improve perinatal outcomes of twin pregnancies.^5^

For decades chorionicity has been determined by US, which is most accurate if performed in the first trimester.^5^ Since 2017, noninvasive analysis of cfDNA using single‐nucleotide polymorphisms (SNP‐based cfDNA) and a proprietary algorithm is a clinically available, validated method to determine zygosity in twin pregnancies, with an accuracy approaching 100%.^6^ SNP‐cfDNA, which analyzes cell‐free fetal DNA from maternal blood as early as 9 weeks of gestation, screens for common chromosomal trisomies, such as 13, 18, and 21, with high sensitivity in twin pregnancies.^6^, ^7^, ^8^, ^9^ Zygosity refers to the genetic similarity of twins. With rare reported exceptions, DZ twins are obligate DC pregnancies.^10^, ^11^, ^12^ Depending upon the timing of embryonic division into twins, MZ twins can present as DC or MC and less commonly as monoamniotic or conjoined twins.^13^, ^14^, ^15^

The accuracy of US examination with respect to chorionicity assignment has been reported to be high (95%–99.8%).^16^, ^17^, ^18^ However, a study by Blumenfeld et al.^19^ compared placental pathology (the gold standard), with US‐determined chorionicity. They reported that even though the average GA of US evaluation was 11.5 weeks, 19% (17/90) of MC twins and 4% (18/455) of DC twins were misclassified by US. The misclassification of an MC pregnancy as DC can result in failure to implement surveillance for complications associated with MC twins, particularly TTTS, with the result being an increased risk for delayed diagnosis/intervention.^20^ Conversely, incorrect assignment of DC twins as MC could lead to heightened surveillance and increased parental anxiety, ultimately resulting in higher healthcare costs and the potential misdiagnosis of pregnancy complications.^20^

This study aimed to compare provider‐assigned chorionicity with zygosity assignment using SNP‐based noninvasive prenatal testing (cfDNA) in a large cohort of twin pregnancies.

## 2 MATERIALS AND METHODS

### 2.1 Inclusion criteria

Twin pregnancy samples were eligible for inclusion if they were obtained in non‐EU countries, had a GA of at least 9.0 weeks, and were received between September 27, 2017 and September 8, 2021. Egg‐donor or surrogate pregnancies, samples that failed quality control, samples with a known vanishing twin and those not receiving zygosity assignment were excluded. All samples were analyzed for zygosity and aneuploidy risk using an SNP‐based cfDNA methodology and algorithms that have been validated previously.^6^, ^8^, ^9^, ^21^, ^22^ Briefly, zygosity was evaluated using DNA sequencing results of 12,568 SNPs. Maternal alleles dominate the cfDNA mixture, allowing confident assignment of the fetal contribution. In MZ pregnancies, a single set of fetal alleles will be identified, while in a DZ pregnancy, there will be discordance within the fetal allele set. The allele distributions within the fetal contribution are tested using the hypothesis that they are from an MZ or a DZ pregnancy and confidence levels are assigned.^6^ Zygosity may be reported for twins when the fetal fraction (FF) is ≥ 1.8%. In DZ twins, both fetuses have an independent FF, and at least one must be ≥ 1.8% for zygosity assignment.

### 2.2 Patient demographics

Maternal demographics, including GA at the time of blood draw, maternal weight, age, ethnicity, and use of in vitro fertilization (IVF) were reported by providers.

### 2.3 Chorionicity analysis

Information regarding the assignment of chorionicity was obtained from the requisition, which included a query using a check box (Supplementary Figure S1). Completion of the check box was not required for analysis. Samples which included an ICD‐10 code specifically related to chorionicity (Supplementary Table S1) were identified and analyzed separately.

### 2.4 Fetal sex distribution analysis

Fetal sex is only reported when requested by the ordering physician and when a minimum FF of ≥2.8% is present (single FF for MZ twins and DZ twins must each have a FF ≥ 2.8%). If the algorithm identifies an MZ pregnancy, fetal sex is only reported if confidence is high that twin fetal sexes are either female/female or male/male. For this analysis, we assumed that all MZ pregnancies would have same‐sex fetuses, 50% female and 50% male. Conversely, for DZ pregnancies, we assumed a sex distribution in which 25% are both male, 25% are both female, and 50% have one male and one female.

### 2.5 Statistical analysis

Pearson chi‐square tests for independence (categorical variables: ethnicity, IVF use, zygosity, and fetal sex) or *z*‐tests for independent samples (continuous variables: GA, maternal age, maternal weight, and FF) were used to examine differences in demographic characteristics between males and females. *p*‐values are not adjusted for multiple comparisons and are intended to be hypothesis‐generating only.

## ETHICAL APPROVAL

This is a retrospective cohort study of cfDNA screening tests from twin gestations received by a Clinical Laboratory Improvement Act (CLIA)‐certified and College of American Pathologists (CAP)‐accredited laboratory (Natera, Inc.; San Carlos, CA, USA). This study was designed in compliance with an investigational review board approved protocol (Ethical and Independent Review Services Study ID, 17,113; date of certification, August 28, 2017, date of renewal August 20, 2020).

## 3 RESULTS

A total of 59,471 twin samples were received, of which 55,344 (93.1%) received a result for zygosity. Zygosity could not be assigned in 6.9% of samples, primarily due to suboptimal sample quality, lab processing errors, and low FF.

Cohort demographics and clinical characteristics are summarized in Table 1. Data are presented for the entire cohort and stratified by zygosity results. The query box on the requisition for chorionicity was completed in 45,236/55,344 (81.7%) of cases, 62.7% of which specified either MC or DC placentation. Table 2 outlines the relationship between zygosity assignment by SNP‐cfDNA and assignment of chorionicity from the requisition. Of the 6283 cases, where MC was checked, 318 (5.1%) were identified as DZ by SNP‐cfDNA. For cases where chorionicity was listed as “don't know”, twin pregnancies were more likely to be MZ when compared to cases where chorionicity was specified (*p* < 0.001).

**TABLE 1:** Cohort demographics

Demographic	Total cohort *N* = 55,344	MZ by SNP‐cfDNA *N* = 16,673	DZ by SNP‐cfDNA *N* = 38,671	*p*‐value*	
GA at draw, completed weeks median, (5^th^, 95^th^ percentile)	12.0 (10.0, 21.0)	12.0 (10.0, 21.0)	12.0 (10.0, 21.0)	*p* > 0.999	
MA (years) median, (5^th^, 95^th^ percentile)	32.0 (22.0, 40.0)	31.0 (21.0, 40.0)	32.0 (23.0, 40.0)	*p* < 0.001	
MW (pounds)^†^ median, (5^th^, 95^th^ percentile)	163.0 (114.0, 266.0)^†^	154.0 (110.2, 243.0)	167 (115.5, 272.0)	*p* < 0.001	
IVF (n/N, %)	538/55,344 (1.0%)	59/16,673 (0.3%)	479/38,671 (1.2%)	*p* < 0.001	
Fetal fraction (%)^#^ median (5^th^, 95^th^ percentile)	11.7% (5.1, 21.7)	11.6% (5.3, 21.3)	11.7% (5.0, 21.9)	*p* = 0.96	

Ethnicity (% of cohort)				MZ versus DZ	All group comparison
White, non‐Hispanic or Latino	22.6%	22.3%	22.7%	*p* = 0.31	*p* < 0.001
Black or African American, non‐Hispanic or Latino	9.1%	6.5%	10.1%	*p* < 0.001	
Hispanic or Latino	7.3%	9.5%	6.4%	*p* < 0.001	
Asian, non‐Hispanic or Latino	1.7%	2.4%	1.3%	*p* < 0.001	
Other, non‐Hispanic or Latino	2.4%	2.8%	2.2%	*p* < 0.001	
Unknown	56.9%	56.4%	57.2%	*p* = 0.082	

*Note*: Demographic information abstracted from requisition for cohort of patients with reported zygosity.

Abbreviations: GA, gestational age, MA, maternal age, MW maternal weight, and ethnicity. Demographic information was analyzed by zygosity and across the total cohort. GA, MA, and MW are reported as median (5th and 95th percentile). *p‐value calculated for comparison of MZ and DZ twins. ^#^Total fetal fraction is combined for DZ twins, a single fetal fraction is reported for MZ twins, and †Cases with reasonable weights were included (70–700 pounds).

**TABLE 2:** Relationship between SNP‐cfDNA zygosity and chorionicity assignment

Chorionicity recorded by provider		Results of SNP‐cfDNA	
		Monozygotic (MZ)	Dizygotic (DZ)
Total cohort	55,344 (100.0%)	16,673 (30.1%)	38,671 (69.9%)
MC	6283 (11.4%)	5965 (94.9%)	318 (5.1%)
DC	28,388 (51.3%)	3927 (13.8%)	24,461 (86.2%)
Listed as “Don't know”	10,565 (19.1%)	3817 (36.1%)*	6748 (63.9%)
Left Blank	10,108 (18.3%)	2964 (29.3%)	7144 (70.7%)

*Note*: *p < 0.001 Percent of MZ cases compared with cases where chorionicity was specified as MC or DC.

A total of 9389/55,344 (17.0%) samples provided an ICD‐10 code that specified information regarding chorionicity (Table 3, Table S1). As described in the tables, these ICD‐10 codes were grouped into four main categories. Of those identified as MC/DA twins, 5.2% were DZ by SNP‐cfDNA. A MC/MA ICD‐10 code was provided in 127/55,344 (1/436) cases. However, using SNP‐cfDNA, 18.9% of these were assigned as DZ twins.

**TABLE 3:** Chorionicity and Zygosity Calls Cross‐Referenced with Chorionicity‐Specific ICD‐10 Codes (if provided)

Chorionicity‐specific ICD‐10 codes	Results of cfDNA SNP‐NIPT	
	Monozygotic (MZ)	Dizygotic (DZ)
Total cohort *N* = 9389	2904 (30.9%)	6485 (69.1%)
Monochorionic/Diamniotic *N* = 807 (8.6%)	765 (94.8%)	42 (5.2%)
Monochorionic/Monoamniotic *N* = 127 (1.3%)	103 (81.1%)	24 (18.9%)
Dichorionic/Diamniotic *N* = 3873 (41.3%)	534 (13.8%)	3339 (86.2%)
Twin pregnancy, unable to determine number of placenta and amniotic sacs *N* = 4582 (48.8%)	1502 (32.8%)	3080 (67.2%)

Abbreviations: DZ, dizygotic; MZ, monozygotic; SNP‐NIPT, single nucleotide polymorphism‐noninvasive prenatal testing.

Given the identified discrepancies between provider‐reported chorionicity and SNP‐cfDNA reported zygosity within our cohort, we performed an exploratory analysis comparing the observed to expected fetal sex distribution for provider‐assigned chorionicity and SNP‐cfDNA assigned zygosity. Only cases where both chorionicity and zygosity were reported were included in this analysis. Fetal sex was requested by the ordering physician in 51,258/55,344 (92.6%) of samples. Fetal sex was reported in 15,409 samples identified as MZ by SNP‐cfDNA (98.8%) and 29,732 samples identified as DZ (83.3%). The primary reason for nonreporting of fetal sex in DZ pregnancies was low FF.

Of the 318 cases for which the provider reported MC, but the SNP‐cfDNA reported DZ, fetal sex was requested by the ordering physician in 296 cases (93.1%) and reported in 231. The observed fetal sex distribution for this group was significantly different from the expected distribution for MZ twins (*p* < 0.001 compared to expected ratio of 50%/50%). Conversely, the observed fetal sex distribution was not significantly different from the expected distribution for DZ twins (female/female, *n* = 64, 27.7%; male/male *n* = 65, 28.1%; female/male, *n* = 102, 44.2%; and *p* = 0.23 compared to expected ratio of 25%/25%/50%). By contrast, for the total cohort, fetal sex distribution was similar to expected in pregnancies assigned as MZ or DZ using SNP‐cfDNA (Table S2).

## 4 DISCUSSION

The key finding in this study is that 1 in 20 cases, either reported as MC by the provider or when an MC specific ICD‐10 code was provided, were assigned as a DZ pregnancy by SNP‐cfDNA. The accuracy of the DZ finding in this cohort is corroborated by the fetal sex distribution in these cases, which is closer to the distribution expected for DZ twins than MZ twins. We are unaware of other studies comparing cfDNA zygosity determination to provider‐reported chorionicity. Historically, zygosity has only been possible using diagnostic testing in pregnancy or postnatally.

Our findings support those reported by Blumenfeld et al.^19^ which compared provider‐assigned chorionicity with histologic reports. These findings are in contrast to other studies,^16^, ^17^, ^18^ where the correlation between US and pathology was found to reach 100%. Although rare cases of MC/DZ twinning have been identified,^10^, ^11^, ^12^ it is unlikely that these rare twin pregnancies would account for the 4%–5% discrepancy in our study.

It is uncertain as to what the current level of understanding is among general obstetrical care providers regarding the genetics of twinning, the importance of an early assignment of chorionicity and the sonographic discriminators between MC and DC twins. In 2004, Cleary‐Goldman et al.^23^ published results of a survey in which 34% of providers believed that chorionicity is best determined in the second trimester. Our data draws attention to the importance of an early assignment of chorionicity and the imprecision of clinical assignment of monochorionicity.

It is expected that MC twin gestations will be managed with more frequent US assessments and testing to monitor fetal well‐being and for signs of TTTS or growth discordance. These pregnancies are more likely to undergo elective early delivery.^24^ Not only can early delivery result in increased healthcare costs, but this is expected to increase anxiety for the family.

A limitation of our study is that information from providers regarding chorionicity was provided in only 62.7% of cases, and we do not know who completed the requisition form. It is possible that errors or omissions of relevant information could be present, even in cases when an ICD‐10 code specific for chorionicity was provided. Perhaps the most objective information suggesting that the discordance in these cases is a “real” finding is the associated lack of correlation with the expected fetal sex distribution for MC pregnancies that were assigned as DZ using SNP‐cfDNA. A further limitation of the study was that the method used for chorionicity assignment was not collected. There are many potential sources of assignment with differences in expected accuracy, including US assessment of chorionicity or fetal sex, the use of preimplantation diagnosis, and placental pathology. Nonetheless, the ability to correlate US and clinical chorionicity assignment with results of SNP‐based zygosity represents an opportunity to reevaluate those cases where discordance is found. Review of clinical information and US findings in the context of a zygosity result may ultimately lead to changes in pregnancy management to optimize the early detection of complications or reduce the need for intensive surveillance.

The failure of twin pregnancies to be diagnosed by midpregnancy^25^ or to reach the second trimester without chorionicity assignment has been recognized.^2^ It is not unusual for twin pregnancies to be identified after the first trimester, even with reliable clinical dating.^26^ Despite guidelines indicating that best performance of US for assignment of chorionicity is prior to 14 weeks, early, high‐quality US prior to 14 weeks gestation is still not common practice.^2^ Baud et al.^20^ reviewed over 300 pregnancies treated with laser ablation for TTTS. In that study, 15% of pregnancies had neither chorionicity assignment prior to referral nor correct assignment of chorionicity. Most importantly, late recognition of correct chorionicity correlated with worse outcomes. Evaluation of zygosity prior to the end of the first trimester and prompt (re)‐evaluation of chorionicity for those assigned to be MZ should lead to increased surveillance and earlier recognition/treatment of TTTS in confirmed MC cases. If DC is confirmed, reduced surveillance can occur.

The American College of Obstetricians and Gynecologists supports the use of cfDNA for twin pregnancy^27^ and recognizes that zygosity can be determined using the SNP‐based method. Our data highlight the need for additional studies to confirm that US assignment of chorionicity, with the adjunct use of SNP‐based screening, will result in more accurate chorionicity determination and related adjustments in management particularly when US outcomes are inconclusive or when abnormalities are found. Indeed, the discrepancy between clinical reporting and cfDNA outcomes are a call to action for clinicians in two major ways. First, we believe our findings support raising awareness and provider education that US assignment of chorionicity is an essential part of the care of twin pregnancies and is most accurately assessed between 9 and 12 weeks.^28^ Second, the use of SNP‐based zygosity assessment should be considered an adjunct for comparison with US, and reevaluations should occur as necessary early in pregnancy. We expect that SNP‐cfDNA zygosity testing combined with sonography will be of significant clinical utility, most importantly by reducing the cost of prenatal care for DZ twins and facilitating earlier intervention and improved outcome for pregnancies at risk for TTTS. For the 30% of MZ twins identified by SNP‐cfDNA, establishing zygosity in the first trimester provides an opportunity for early referral to maternal–fetal medicine specialists for better delineation of placentation. Increased surveillance in specialized centers is beneficial for those women at highest risk for complications from MC placentation.

Further research should determine the impact of the addition of first trimester zygosity assignment for twin pregnancies upon the accuracy of chorionicity assignment, and the differences in healthcare costs for pregnancies assigned either MZ or DZ genetic origin. Finally, there is limited information on the impact of zygosity (corrected for chorionicity) upon pregnancy outcome. Our study lays a foundation for such research, to better determine the degree to which these two factors contribute independently to complicated and normal outcomes.

## CONFLICT OF INTEREST

Andrei Rebarber is a speaker for Natera, Inc. and Hologic. Kimberly A. Martin is a consultant to Natera, Inc. with stock/options to own stock in the company. Russ Jelsema, Allyson Koyen Malashevich, Katelyn Hashimoto, Sheetal Parmar, Zachary Demko, and Paul Billings, are employees of Natera, Inc. with stock/options to own stock in the company.

## ETHICS STATEMENT

This study was designed in compliance with an investigational review board approved protocol (Ethical and Independent Review Services Study ID, 17,113; date of certification, August 28, 2017, date of renewal August 20, 2020). Written informed consent was obtained from all study participants.

## COPYRIGHT STATEMENT

The authors confirm that this manuscript represents original research that has not been previously published nor being considered for publication elsewhere.

## Supporting information

Supporting Information S1

## Data Availability

The data that support the findings of the study are available upon request to the corresponding author (Andrei Rebarber) for consideration. The data are not publicly available due to privacy or ethical restrictions.
