# Supplementary material for: Clinician‐reported chorionicity and zygosity assignment using single‐nucleotide polymorphism‐based cell‐free DNA: Lessons learned from 55,344 twin pregnancies
Source: Prenat Diagn. 2022 Sep 7;42(10):1235–41. doi: 10.1002/pd.6218 (PMC9541063; doi:10.1002/pd.6218)
Supplement: Supplementary file 1 — Supporting Information S1 [file PD-42-1235-s001.docx]

**SUPPLEMENTARY INFORMATION**

***Original Manuscript***

**Title:** Clinician-reported Chorionicity and Zygosity Assignment using single-nucleotide polymorphism-based cell-free DNA: Lessons learned from 55,344 Twin Pregnancies

**Authors:** Ms. Anna Wojas, B.S.^1^, Dr. Kimberly A. Martin, M.D.^2^, Dr. Allyson Koyen Malashevich, Ph.D.^2^, Ms. Katelyn Hashimoto, M.S.^2^, Ms. Sheetal Parmar, M.S.^2^, Ms. Roseann White, M.A.^2^, Dr. Zachary Demko, Ph.D.^2^, Dr. Paul Billings, M.D., Ph.D.^2^, Dr. Russ Jelsema, M.D.^2^, and Dr. Andrei Rebarber, M.D.^1^

**Affiliations:**

1. Department of Obstetrics, Gynecology, and Reproductive Science, Mt. Sinai, New York, NY, USA

2. Natera Inc., Austin, TX, USA

**Corresponding Author:**

**Andrei Rebarber, M.D.**

Clinical Professor

Department of Obstetrics, Gynecology, and Reproductive Science

Icahn School of Medicine at Mount Sinai

New York, NY

Email: arebarber@mfmnyc.com

**Figure S1: Requisition form**

**
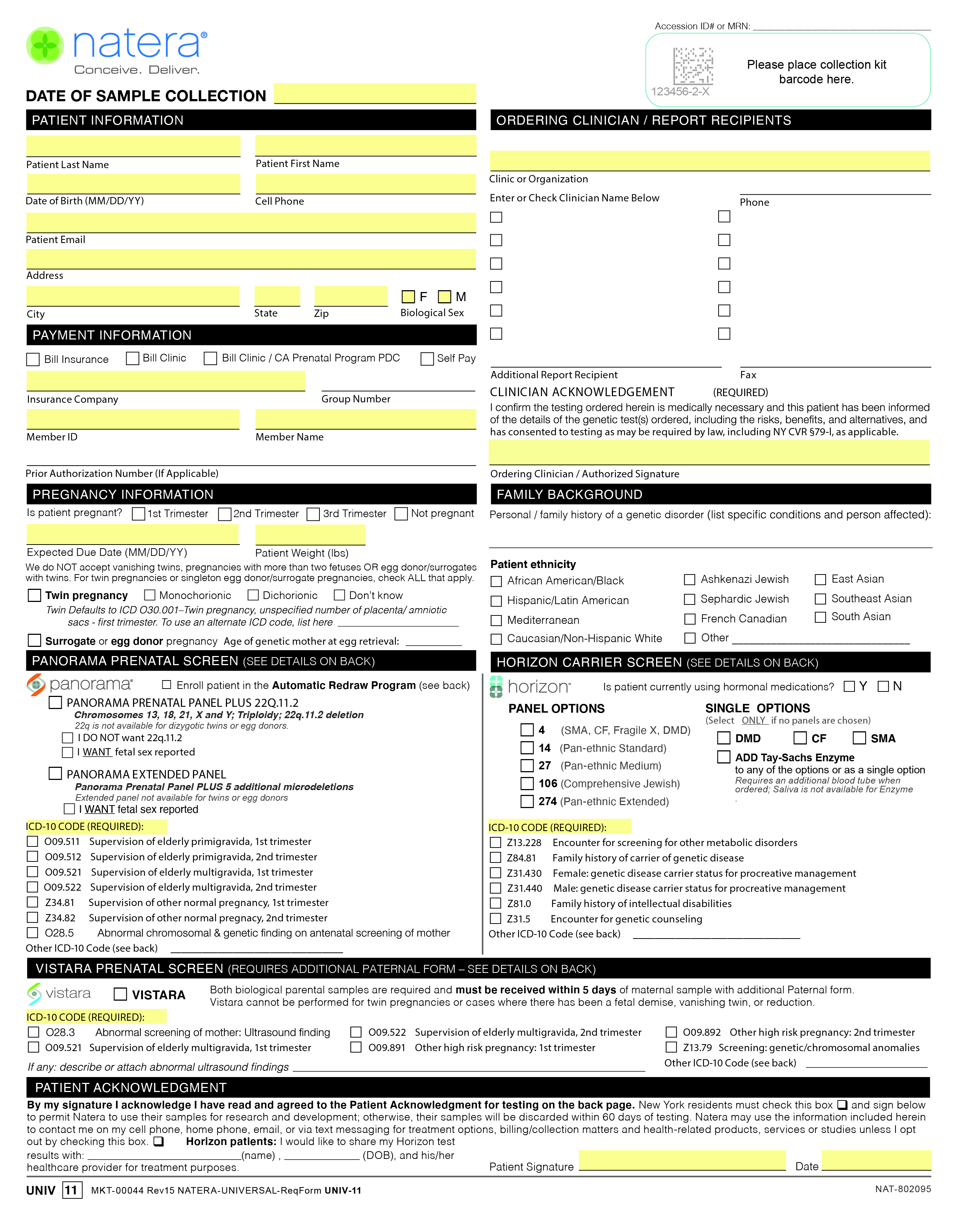
**

**Figure S1:** Snapshot from requisition form used to order NIPT by providers. Twin pregnancy and chorionicity status could be recorded by check box. Additionally, providers had the option to record relevant ICD codes in the open field.

**Table S1. ICD-10 Chorionicity-Related Codes for Subanalysis**

| ICD Code Indication | Number of Cases | ICD-10 Codes |
| --- | --- | --- |
| Monochorionic/  Diamniotic | 807 | O30.03, O30.031, O30.032, O30.033, O30.039 |
| Monochorionic/  Monoamniotic | 127 | O30.011, O30.012, O30.019 |
| Dichorionic/  Diamniotic | 3,873 | O30.04, O30.041, O30.042, O30.043, O30.049 |
| Twin pregnancy, unable to determine number of placenta and number of amniotic sacs | 4,582 | O30.00, O30.001, O30.002, O30.003, O30.009, O30.091, O30.092, O30.093, O30.099 |

**Table S1.** ICD-10 codes were reviewed for the entire cohort. Four code-sets specific to amnionicity and chorionicity were identified as described in this table.

**Table S2:** Fetal sex distribution by chorionicity/zygosity for twin cases with both sexes reported

|  | SNP-cfDNA MZ N= 15,409 | SNP-cfDNA DZ  N=29,732 |
| --- | --- | --- |
| Fetal Sex Distribution | female/female n= 7,893 (51.2%)  male/male n= 7,516 (48.8%) | female/female n= 7,251 (24.4%)  male/male n= 7,629 (25.7%)  female/male n= 14,852 (50.0%) |

**Table S2:** MZ, monozygotic; DZ, dizygotic; MC, monochorionic; DC
